# Refractory Hypoglycaemia Caused by a Pleuropulmonary Solitary Fibrous Tumour: A Case of Doege-Potter Syndrome

**DOI:** 10.7759/cureus.77294

**Published:** 2025-01-11

**Authors:** Lyndon Sprenghers, Kathleen Bollaerts, Annelies Leyssens, Anne-Marie Vandenbroucke, Michel Vandenbroucke

**Affiliations:** 1 Department of Pulmonary Medicine, KU Leuven, Leuven, BEL; 2 Department of Endocrinology, AZ Sint-Maarten, Mechelen, BEL; 3 Department of Pulmonary Medicine, AZ Sint-Maarten, Mechelen, BEL

**Keywords:** diaphragm elevation, doege-potter syndrome, hypoglycaemia, paraneoplastic syndrome, solitary fibrous tumours

## Abstract

Hypoglycaemia has a very broad differential diagnosis. It is important to perform a thorough examination of the possible causes. Doege-Potter syndrome is a rare paraneoplastic syndrome that is associated with refractory hypoglycaemia and is caused by solitary fibrous tumours (SFTs). Diagnosis can be suspected based on imaging and clinical presentation, but the definitive diagnosis requires histologic confirmation. Most SFTs are located in the thorax. Therefore, imaging plays a major role in the diagnosis. CT scan should be performed when diaphragm elevation is seen on X-ray to exclude underlying masses and to make the differential diagnosis with diaphragm paralysis. Treatment of SFT is still not established, and no clear guidelines are available. In this case report, we describe the case of a 65-year-old woman who presented with refractory hypoglycaemia caused by a thoracic SFT.

## Introduction

Doege-Potter syndrome is a rare paraneoplastic syndrome caused by solitary fibrous tumours (SFTs) and presents with hypoglycaemia. It is caused by tumour secretion of insulin-like growth factor 2 (IGF-2). Refractory hypoglycaemia occurs in less than 5% of cases and is seen more frequently in large tumours. SFTs are a rare and heterogeneous group of fibroblastic mesenchymal neoplasms with variable behaviour, ranging from benign to malignant. They rarely metastasise. SFTs are most frequently seen in the fifth to seventh decades of life but may arise at any age. SFTs arise in the thorax in 50%-70% of cases, predominantly from the visceral pleura [1]. Clinical presentation depends greatly on the anatomic site of the tumour. Pleuropulmonary SFT can present with cough, shortness of breath, or chest pain [2]. Haemoptysis is rare. Doege-Potter syndrome presents as refractory hypoglycaemia [3]. It is classified by the endocrine society as a form of fasting hypoglycaemia; other causes of hypoglycaemia should be excluded. SFTs are part of a group of tumours causing hypoglycaemia via the secretion of IGF-2 (non-islet cell tumour-induced hypoglycaemia (NICTH)). Diagnosis can be suspected based on imaging and clinical presentation, but the definitive diagnosis requires histologic confirmation. Treatment for localised disease is complete surgical resection. Radiotherapy has a role in selected cases. Treatment for locally advanced disease and metastatic disease is not established and can consist of targeted therapy, radiotherapy, or chemotherapy. In this case report, we describe the case of a 65-year-old woman who presented with refractory hypoglycaemia caused by a thoracic SFT. This case is an example of the importance of early detection and management of SFT, as this syndrome could have been avoided by earlier resection of the tumour (retrospectively, the diagnosis of a small SFT was already made in the past).

## Case presentation

Patient description

We present the case of a 65-year-old woman who presented to the emergency department because of weird behaviour. The patient lived alone and was taken to the hospital by her neighbours. In 2013, she underwent a broad excision for ductal adenocarcinoma of the left breast. She received adjuvant radiotherapy and was still using tamoxifen. She was also in follow-up because of a myoma of the uterus. She did not have any other relevant history.

Case history

The patient was taken to the hospital because of odd behaviour for a few days: she had been confused, suffered day-night reversal, and was showing disinhibited behaviour. She also described disturbed vision. The behaviour normalised completely after drinking a sugary drink. There were no other complaints. A thorough history was taken after the diagnosis of hypoglycaemia, 27 mg/dL (reference values 60-110 mg/dL), at the emergency department. The patient lived alone; she was not known to have diabetes and did not know anyone who used insulin. She only drank alcohol sporadically. She denied smoking or other drugs. There were no known liver problems or any other diseases. She did use vitamin supplements and fish oil. She was on a sugar-free diet but did not completely ban carbohydrates. She did not have any other symptoms. During her hospitalisation, she did have recurrent episodes of hypoglycaemia, and we noticed an important hypoglycaemia unawareness.

Physical examination results

Initial clinical evaluation was unremarkable, except for an orbital haematoma. Auscultation of the lungs was normal. Neurological examination was normal. As mentioned earlier, no clinical abnormalities were noticed during the episodes of hypoglycaemia (unawareness). The patient did have hypertension during hospitalisation.

Results of pathological tests and other investigations

**Figure 1:**
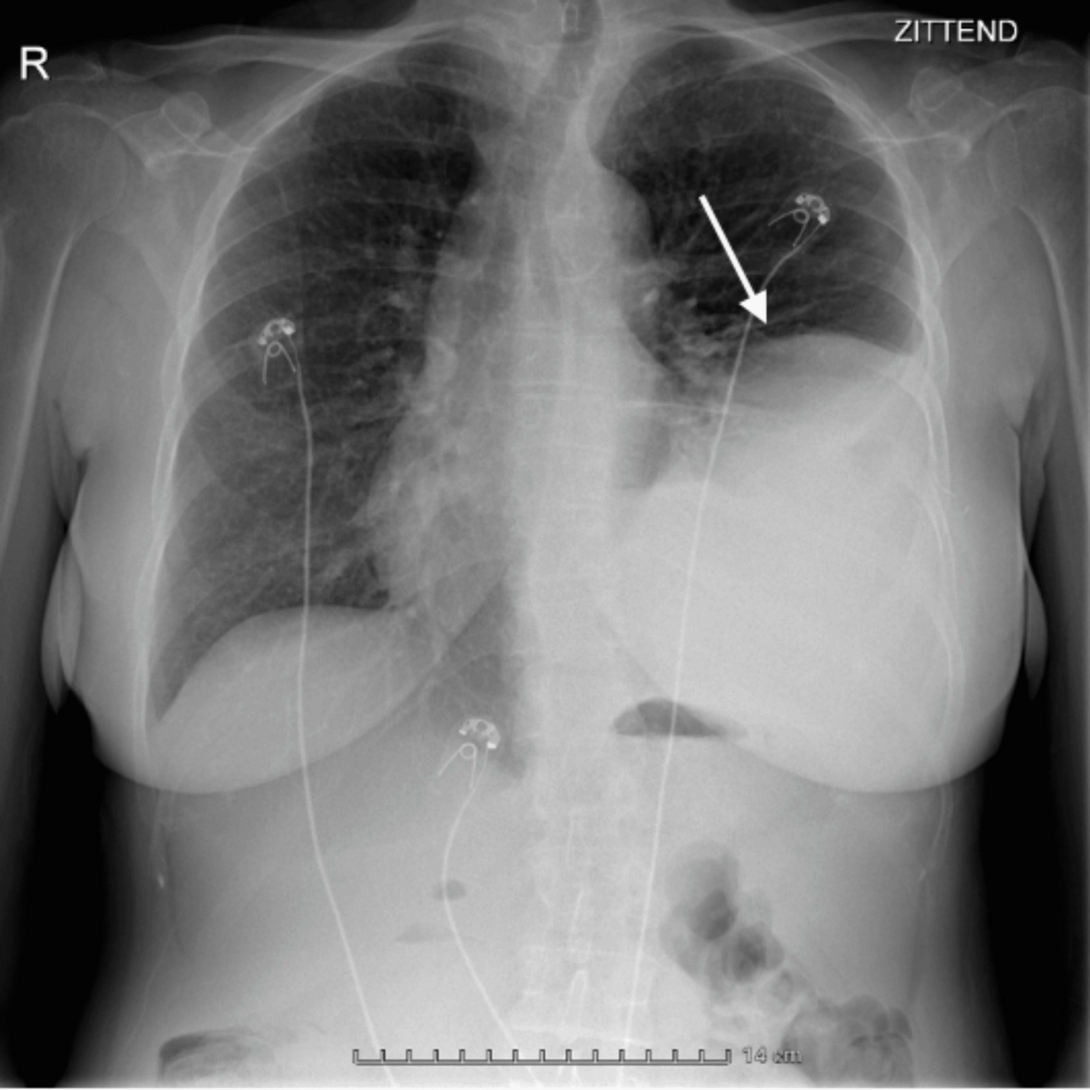
Chest X-ray on admission showed elevation of the left hemidiaphragm.

Initial lab results at the emergency department showed a hypoglycaemia of 27 mg/dL (reference values 60-110 mg/dL), hypokalaemia, and hypernatraemia. There were no signs of infection, no marked abnormalities in the liver function tests, and normal coagulation tests. C-peptide was suppressed (<0.01 nmol/L, reference values 0.270-1.270 nmol/L). Chest radiography showed an opacity obscuring the left hemidiaphragm and left heart border, with a mild shift of the trachea to the right and a pleural effusion (Figure 1).

**Figure 2:**
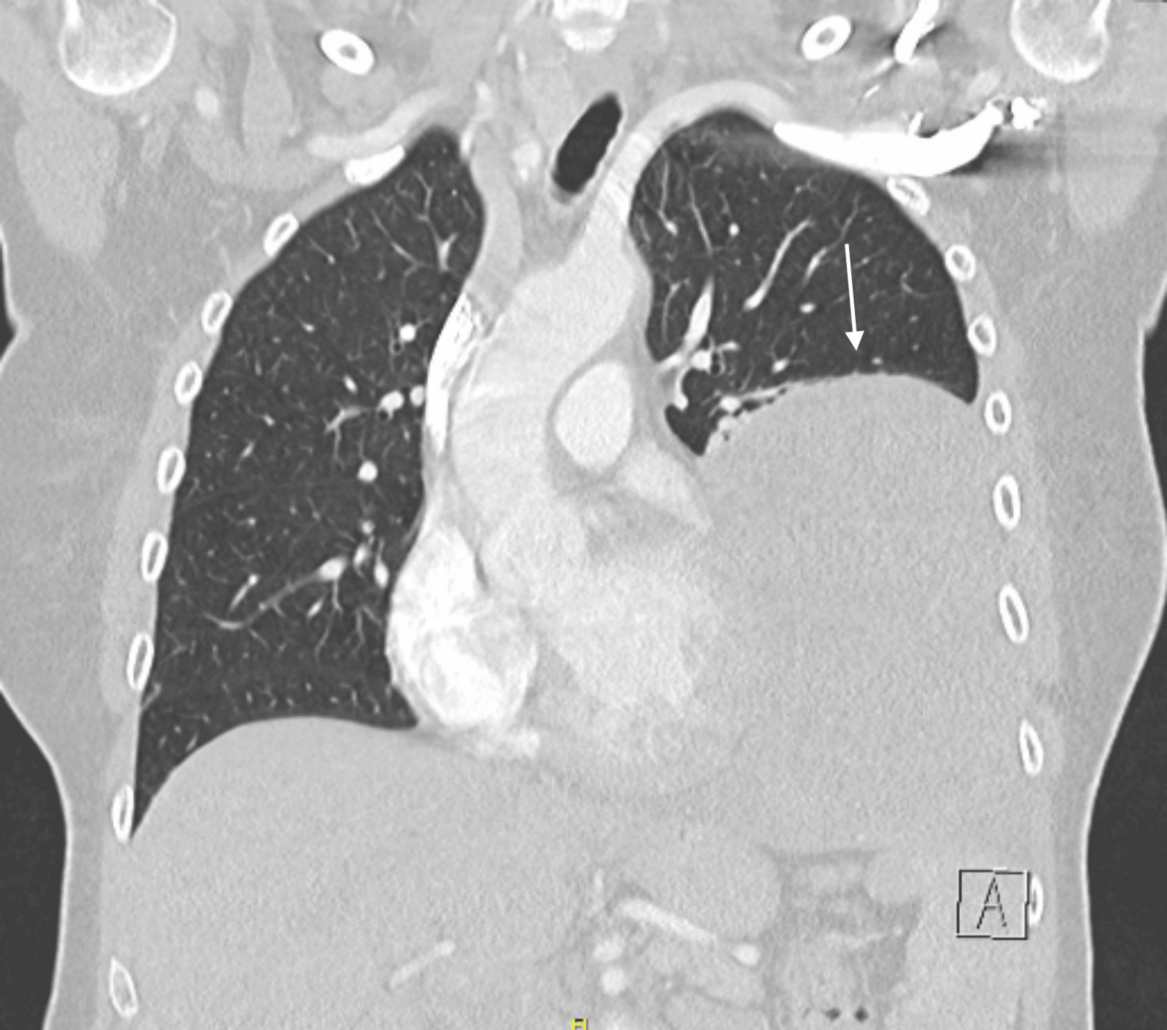
CT of the thorax showed a mass in the lower lobe of the left lung. Coronal plane, lung window.

**Figure 3:**
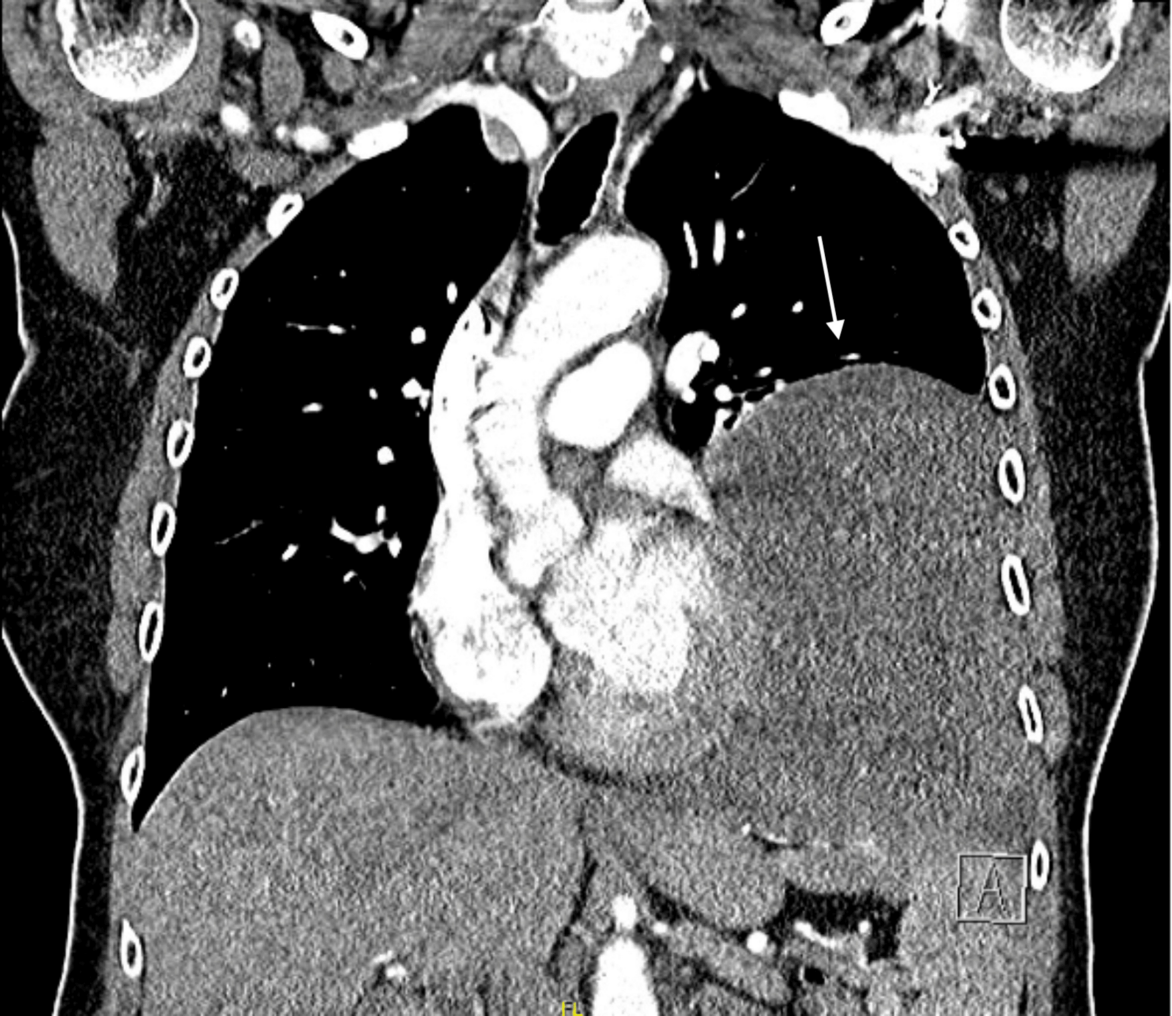
CT of the thorax showed a mass in the lower lobe of the left lung. Coronal plane, soft tissue window.

Because of the confusion, a CT of the brain was performed, which was normal. During hospitalisation, the patient developed recurrent episodes of hypoglycaemia, as low as 36 mg/dL (reference values 60-110 mg/dL), while receiving intravenous glucose. C-peptide and insulin were checked twice during an episode of hypoglycaemia. Both controls showed a suppressed C-peptide (0.03 and 0.04 nmol/L, reference values 0.270-1.270 nmol/L) and low insulin (1.728 mU/L, reference values 3-25 mU/L). GAD65 antibodies were negative. Pituitary axes were checked and were normal. Because of the electrolyte disturbances (sodium and potassium), a 24-hour urinary cortisol collection was performed, showing no cortisol excess. We performed these tests to exclude other causes of hypoglycaemia, for example, insulinoma (C-peptide and insulin) and adrenal disease. Because of the abnormalities on chest radiography, we performed a CT of the thorax (Figures 2, 3).

**Figure 4:**
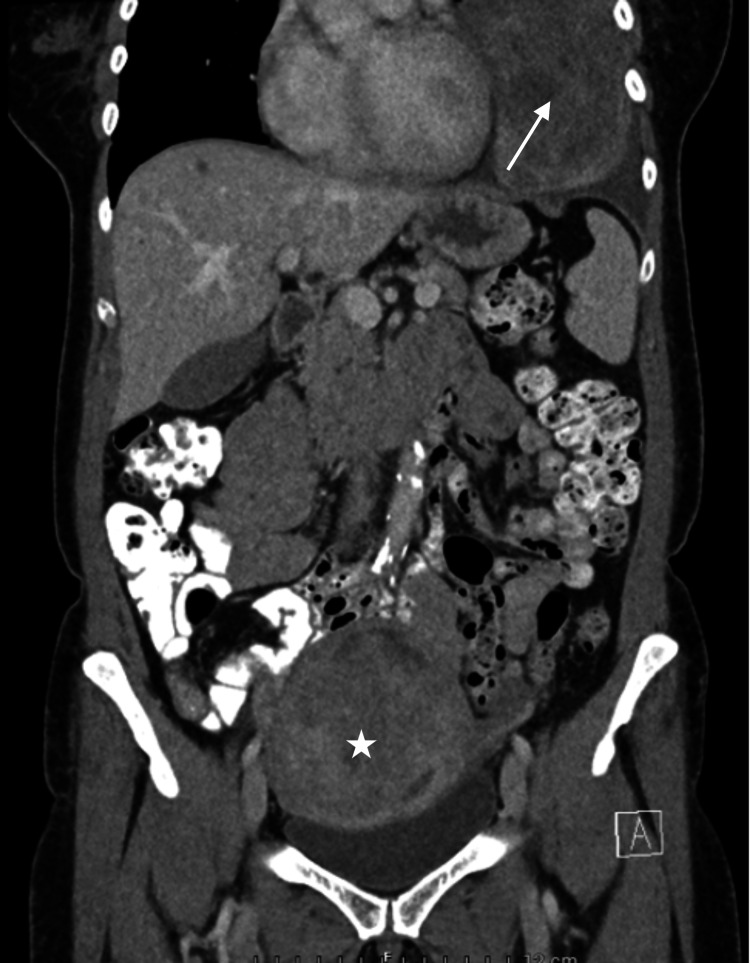
CT of the abdomen showed a uterine mass. No metastases. Coronal plane. White star: uterine mass; white arrow: pleuropulmonary solitary fibrous tumour (SFT)

CT scan shows the presence of a sharply delineated low-density solid tissue mass in the left lower lung lobe, with visible internal vascularisation. The mass measures 13 cm anteroposteriorly, 11 cm transversely, and occupies the entire left lower lung zone. Secondary passive atelectasis of the left lower lobe and fibrotic changes in the lingula are noted. Lung window images show no associated micronodules in the upper lobe or in the right lung. Imaging was consistent with a schwannoma or a SFT. We also performed a CT of the abdomen to rule out metastatic disease. This showed a normal pancreas, a haemangioma of the liver, and a uterine mass for which the patient was referred to the gynaecology department (Figure 4).

Retrospectively, we found a CT scan from 2013 that showed a small mass in the lower lobe of the left lung (3.4 cm × 3 cm). The CT was performed in the context of follow-up for the breast carcinoma. At the time, a PET scan was performed; the mass was not FDG-avid. A biopsy was performed and showed a SFT.

After excluding other causes of hypoglycaemia (liver disease, infection, insulinoma, new-onset diabetes mellitus type 1, insulin use, adrenal insufficiency, …), we found cases in the literature about paraneoplastic syndromes that cause hypoglycaemia. The diagnosis of Doege-Potter syndrome was suspected. For the definitive diagnosis, we needed histologic confirmation. We did not perform a biopsy because the type of tumour was not known yet, and because of the potential risk of tumour soiling, this decision was made after consulting our oncologists and thoracic surgeons. The patient was referred for surgery. We were not able to determine IGF-2 in our hospital; this could have confirmed the pathophysiology of the Doege-Potter syndrome.

Treatment plan

**Figure 5:**
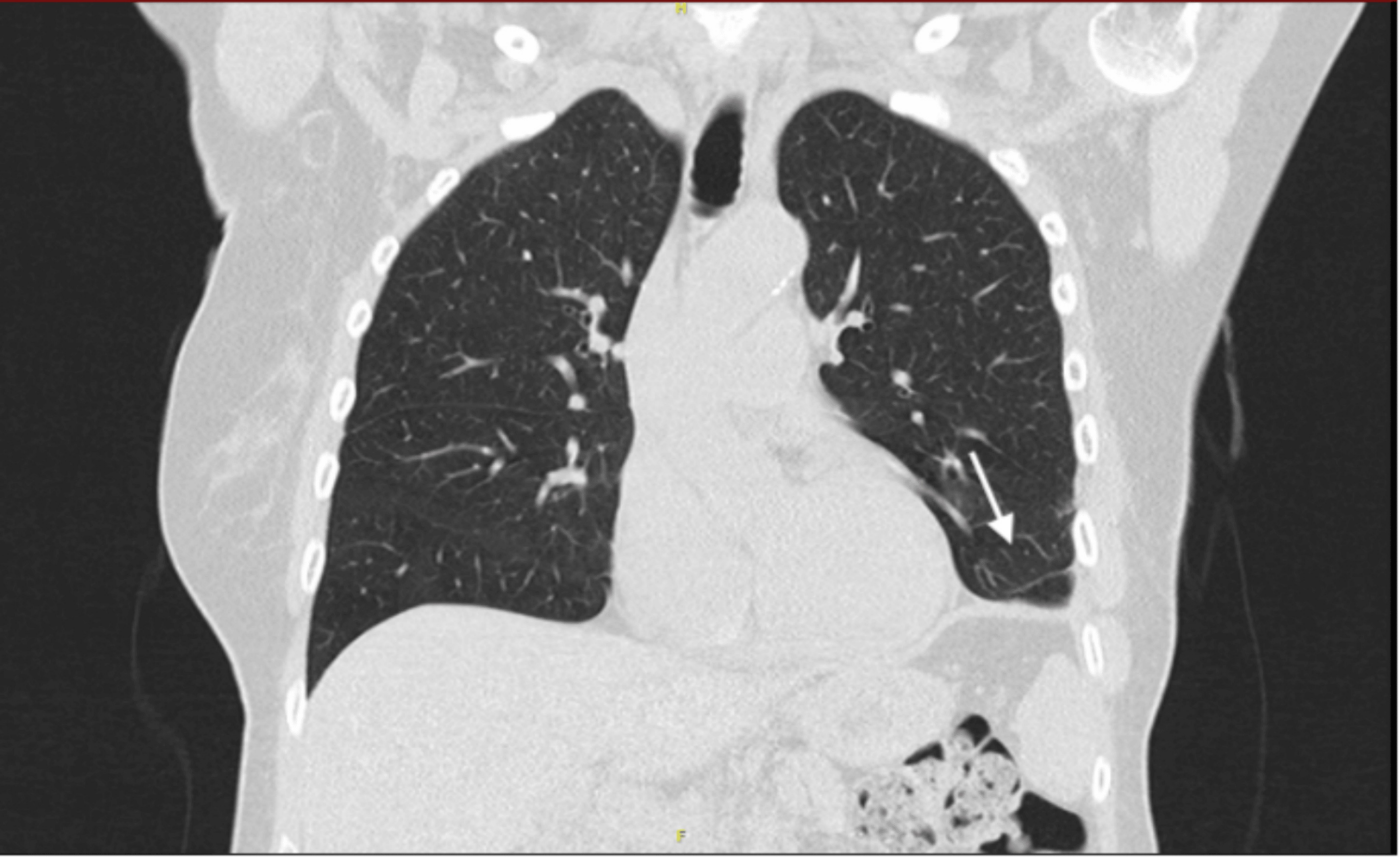
CT of the chest showing complete resection of the mass. Coronal plane, lung window.

The patient was referred to a tertiary centre for surgery. Because of the repeated deep hypoglycaemia during hospitalisation, the patient was started on dexamethasone 8 mg per day. With this treatment, the blood glucose was within normal ranges. The tumour was excised via left anterior thoracotomy, together with part of the parietal pleura. The surgery was uncomplicated, and the patient recovered quickly. CT of the chest showed complete resection (Figure 5).

Pathologic examination of the resected tumour did indeed confirm the diagnosis of a SFT, with tumour-free margins of at least 0.3 cm. Pathologic examination of the parietal pleura showed no signs of malignancy. Because of the absence of metastatic disease and the complete resection of the tumour, no adjuvant therapy was started.

Expected outcome of the treatment plan

Because of the histologic confirmation, we were convinced that the hypoglycaemia was based on the Doege-Potter syndrome. We would expect that the blood glucose levels would return to normal after the resection of the tumour.

Actual outcome

During the first days after surgery, the patient had already developed hyperglycaemia. At that moment, she was still taking dexamethasone. Dexamethasone was stopped, and blood glucose levels remained normal. She was discharged six days after surgery; at the time, glycaemia was normal. One month after surgery, she was seen at the endocrinology consultation, which showed normal blood glucose levels; the patient had already stopped measuring blood glucose. We conclude that the hypoglycaemia was caused by the Doege-Potter syndrome because of the clear temporal relationship between the resection of the tumour and the normalisation of the blood glucose levels. Because of the risk of recurrence, follow-up was planned.

## Discussion

Doege-Potter syndrome is a rare paraneoplastic syndrome related to SFTs. It is classified by the endocrine society as a form of fasting hypoglycaemia.

Many tumours have been associated with this type of paraneoplastic syndrome. It is referred to as NICTH [4]. The hypoglycaemia is caused by the overexpression of IGF-2; this results in an increase of circulating precursors of the IGF-2 protein. This, in turn, will lead to a change in levels of insulin and other proteins (IGF-1, IGF-binding proteins, …) causing hypoglycaemia.

Because it is a rare cause of hypoglycaemia, a thorough examination should be performed to rule out other causes of hypoglycaemia (infection, liver failure, exogenous insulin, …). The measurement of C-peptide and insulin at the time of hypoglycaemia can already make an important differentiation, for example, for the diagnosis of insulinoma.

Imaging should be performed to rule out any malignancies in cases of persisting hypoglycaemia with no clear aetiology. In our case, the chest X-ray made at the emergency department showed an opacity obscuring the left hemidiaphragm and left heart border, with a mild shift of the trachea to the right and a pleural effusion. Elevation of the hemidiaphragm can be caused by diaphragm paralysis or by mechanical effects of focal pathology.

The differential diagnosis between elevation of the diaphragm and diaphragm paresis should be made because the causes are different. As in most cases, this was an incidental finding. The chest X-ray was made in the emergency department as part of screening because of the deep hypoglycaemia. In addition to a thorough anamnesis and clinical examination, the next step in making the differentiation between elevation and paresis is a CT scan. CT scan excludes the causes of diaphragm elevation, e.g., subpleural effusion or mass. In our case, this showed the added mass. If the CT scan is normal, a more elaborate workup with a sniff test, pulmonary function testing, and lab tests should be performed [5].

The diagnosis of Doege-Potter syndrome can only be made after histologic confirmation. It is, however, not always the best option to perform a biopsy because of the risk of soiling or the risks associated with performing a biopsy on a secreting tumour [6]. In our case, there was a quite broad differential diagnosis for the type of tumour based on clinical and radiological presentation. For this reason, we referred our patient to a tertiary centre for complete surgical resection. As explained earlier, Doege-Potter syndrome is caused by the overexpression of IGF-2. In our centre, the execution of this analysis was not possible. The analysis of IGF-2 plays an important role in the diagnosis of Doege-Potter syndrome, as it is directly linked to the pathophysiology. However, we did conclude that the hypoglycaemia was caused by Doege-Potter syndrome because of the clear temporal relationship between the resection of the tumour and the normalisation of the blood glucose levels. Some cases of persisting hypoglycaemia with the necessity for long-term treatment have been documented. These cases all involved metastatic disease where complete surgical resection was not possible [4].

Due to the lack of randomised controlled trials, there is no uniform treatment strategy for SFT [7]. The standard of care for localised disease is complete resection to negative margins. For adjuvant therapy, no clear guidelines are available. It is recommended to discuss these cases with a multidisciplinary team. The best management for locally advanced and metastatic disease is also not established. In our case, the diagnosis of SFT was already made in 2013, with histologic confirmation at the time. Nevertheless, our patient did not get any follow-up or treatment; the tumour was labelled as benign. We did not find any guidelines about the follow-up of this type of tumour; given the fact that they have malignant potential, we believe that early treatment is best. Smaller tumours are more likely to be resected completely and can be resected in a less invasive way.

We did find positive advice for routine follow-up after treatment because of the possibility of recurrence. There are no guidelines on how this surveillance should be done, but there is a need for lifetime surveillance because recurrence can occur many years after diagnosis and treatment. In this case, follow-up of the SFT in 2013 might have prevented the development of Doege-Potter syndrome.

## Conclusions

Hypoglycaemia has a very broad differential diagnosis ranging from very common causes, such as exogenous insulin use, to rare causes, such as paraneoplastic syndromes. It is important to perform a thorough examination of the possible causes. Doege-Potter syndrome is a rare paraneoplastic syndrome that is associated with refractory hypoglycaemia and is caused by a SFT. Diagnosis can be suspected based on imaging and clinical presentation, but the definitive diagnosis requires histologic confirmation. Treatment is not established by guidelines, but complete resection is the standard of care for localised disease. There is certainly a need for clearer guidelines on the treatment and follow-up of SFT. Elevation of the diaphragm should always be investigated, as it can be caused by underlying masses or other potentially harmful diseases.
